# The association of preoperative cardiac stress testing with 30-day death and myocardial infarction among patients undergoing kidney transplantation

**DOI:** 10.1371/journal.pone.0211161

**Published:** 2019-02-01

**Authors:** Tim Dunn, Mohammed J. Saeed, Adam Shpigel, Eric Novak, Tarek Alhamad, Dustin Stwalley, Michael W. Rich, David L. Brown

**Affiliations:** 1 Cardiovascular Division, Washington University School of Medicine, St. Louis, MO, United States of America; 2 Department of Internal Medicine, Washington University School of Medicine, St. Louis, MO, United States of America; 3 Department of Internal Medicine, Renal Division, Washington University School of Medicine, St. Louis, MO, United States of America; Beth Israel Deaconess Medical Center, UNITED STATES

## Abstract

**Background:**

Although periodic cardiac stress testing is commonly used to screen patients on the waiting list for kidney transplantation for ischemic heart disease, there is little evidence to support this practice. We hypothesized that cardiac stress testing in the 18 months prior to kidney transplantation would not reduce postoperative death, total myocardial infarction (MI) or fatal MI.

**Methods:**

Using the United States Renal Data System, we identified ESRD patients ≥40 years old with primary Medicare insurance who received their first kidney transplant between 7/1/2006 and 11/31/2013. Propensity matching created a 1:1 matched sample of patients with and without stress testing in the 18 months prior to kidney transplantation. The outcomes of interest were death, total (fatal and nonfatal) MI or fatal MI within 30 days of kidney transplantation.

**Results:**

In the propensity-matched cohort of 17,304 patients, death within 30 days occurred in 72 of 8,652 (0.83%) patients who underwent stress testing and in 65 of 8,652 (0.75%) patients who did not (OR 1.07; 95% CI: 0.79–1.45; P = 0.66). MI within 30 days occurred in 339 (3.9%) patients who had a stress test and in 333 (3.8%) patients who did not (OR 1.03; 95% CI: 0.89–1.21; P = 0.68). Fatal MI occurred in 17 (0.20%) patients who underwent stress testing and 15 (0.17%) patients who did not (OR 0.97; 95% CI: 0.71–1.32; P = 0.84).

**Conclusion:**

Stress testing in the 18 months prior to kidney transplantation is not associated with a reduction in death, total MI or fatal MI within 30 days of kidney transplantation.

## Introduction

Cardiovascular disease is a major cause of morbidity and mortality for patients with end stage renal disease (ESRD) on the waiting list for kidney transplantation and is the leading cause of death after kidney transplantation [1–5]. Thus, reducing cardiovascular mortality after kidney transplantation is critically important as donor kidneys are a limited resource that should not be allocated to patients who are at high risk of potentially fatal perioperative cardiovascular events.

Because patients with ESRD have higher rates of cardiovascular disease than the general population, especially ischemic heart disease, screening for ischemia prior to placing patients on the transplant waiting list is performed on most adult patients [6]. Patients without ischemia and other contraindications to transplant are placed on the waiting list. Patients with ischemia are commonly referred for coronary angiography and, if obstructive coronary artery disease is present, undergo revascularization with percutaneous coronary intervention (PCI) or coronary artery bypass graft surgery (CABG) after which they are placed on the waiting list. Patients with extensive disease that is not amenable to revascularization are usually excluded from consideration of transplant and never placed on the waiting list [7].

Since the timing of donor availability and transplant surgery is unpredictable, asymptomatic patients with ESRD who have already been screened for ischemia prior to placement on the waiting list commonly undergo stress testing [6] to identify and revascularize those who have developed ischemia since their initial screening for transplant candidacy. One common strategy is to perform annual stress tests while the patient is on the waiting list. However, the lack of clinical evidence supporting subsequent stress testing in patients already evaluated for ischemia prior to placement on the waiting list for kidney transplant has led to varying consensus recommendations and considerable practice variation across transplant centers [8]. Given that in other clinical settings there is no evidence that assessing for ischemia or revascularization in asymptomatic patients pre-operatively improves post-operative outcomes, we hypothesized that surveillance of patients with ESRD who are on the waiting list for kidney transplantation with cardiac stress testing in the 18 months prior to surgery would not be associated with a reduction in perioperative (30-day) death, total (fatal and nonfatal) myocardial infarction (MI) or fatal MI.

## Methods

### Data source

Data on patients undergoing renal transplantation were obtained from the USRDS (United States Renal Data System) database. The USRDS is a comprehensive data set that includes information on all patients in the United States who develop ESRD and who require renal replacement therapy, either dialysis or renal transplantation. The USRDS is funded by the National Institute of Diabetes and Digestive and Kidney Diseases (NIDDK). The USRDS includes *International Classification of Diseases*, *Ninth Revision*, *Clinical Modification* (ICD-9-CM) diagnosis and procedure codes, and Current Procedural Terminology, 4th Edition (CPT-4) codes for Medicare services and transplant information from the United Network for Organ Sharing (UNOS).

### Study population

The study population included ESRD patients ≥40 years of age who underwent their first kidney transplant between 7/1/2006 and 11/31/2013. The study population was limited to patients who were insured by Medicare parts A and B for at least 18 months prior to kidney transplantation. Patients were excluded if no claims were made in the 18 months prior to transplant surgery. Stress tests were identified from ICD-9-CM or CPT-4 codes and included exercise and pharmacologic stress. To eliminate patients who may have undergone coronary angiography instead of stress testing as well as those with active ischemic heart disease, individuals were excluded if coronary angiography, PCI or CABG without stress testing or prior to stress testing were performed in the 18 months prior to transplantation.

Baseline characteristics including age, sex, height, weight, race and comorbidites were recorded for the entire study population. Comorbidities were recorded using the Elixhauser comorbidity classification which is a method of categorizing comorbidities based on ICD-9-CM codes found in administrative data [9]. Missing height and weight data (each <2%) were imputed using a sequential imputation algorithm. A regression predictive mean matching method was employed; all available data were used when developing regression models for height and weight imputation.

Primary outcomes of interest included death from any cause within 30 days following renal transplant surgery, total MI (fatal and nonfatal) within 30 days of surgery or fatal MI within 30 days of surgery. MI was defined by ICD-9-CM codes 410.xx. Fatal MI was defined by an MI following surgery and death within 30 days of the MI.

### Statistics

Comparisons of patient characteristics between those with and without stress testing were performed using Student’s two sample t-test for continuous variables and chi-square test for categorical data. All non-normal and ordinal data were summarized by median (1^st^ quartile, 3^rd^ quartile) and compared using Mann-Whitney U-test. A logistic regression model was created to identify the propensity for stress testing using the following variables: age, sex, transplant year, race, body mass index (BMI), donor type, primary disease for ESRD, chronic heart failure, heart valve disease, pulmonary circulation disease, peripheral vascular disease, paralysis, other neurological disorders, chronic lung disease, diabetes, hypertension, hyperthyroidism, liver disease, peptic ulcer disease, acquired immune deficiency syndrome, lymphoma, metastatic cancer, solid tumor without metastasis, rheumatoid arthritis, coagulopathy, fluid and electrolyte disorders, chronic blood loss anemia, deficiency anemias, psychoses, depression, history of MI, CAD, pericardial disease, endocarditis, cardiomyopathy, cerebrovascular disease, drug abuse, alcohol abuse, weight loss, arrhythmia, cardiac arrest/ventricular fibrillation, tobacco use, lipid metabolism disorder, and transplant center volume. A Greedy matching algorithm was then used to create a 1:1 propensity score-matched sample. Standardized differences were calculated to examine covariate balance before and after matching.

Logistic regression analysis was performed to analyze the association between stress testing and the outcomes of MI, death and fatal MI in the 30-day postoperative period for the total sample and for the propensity score-matched sample. For the total sample, multivariable models were created that included all the same variables used to build the propensity score as independent adjustment variables. For the propensity score matched sample, a mixed model approach was used to account for the matching. Results from both the propensity score matching and multivariable regression analyses are provided separately. All models treated center as a random effect to account for practice variation across transplant facilities. All analyses were performed using SAS version 9.3. Two authors (MJS, EN) had full access to the data and assume responsibility for the integrity of the data and analyses performed.

## Results

### Study population

**Fig 1:**
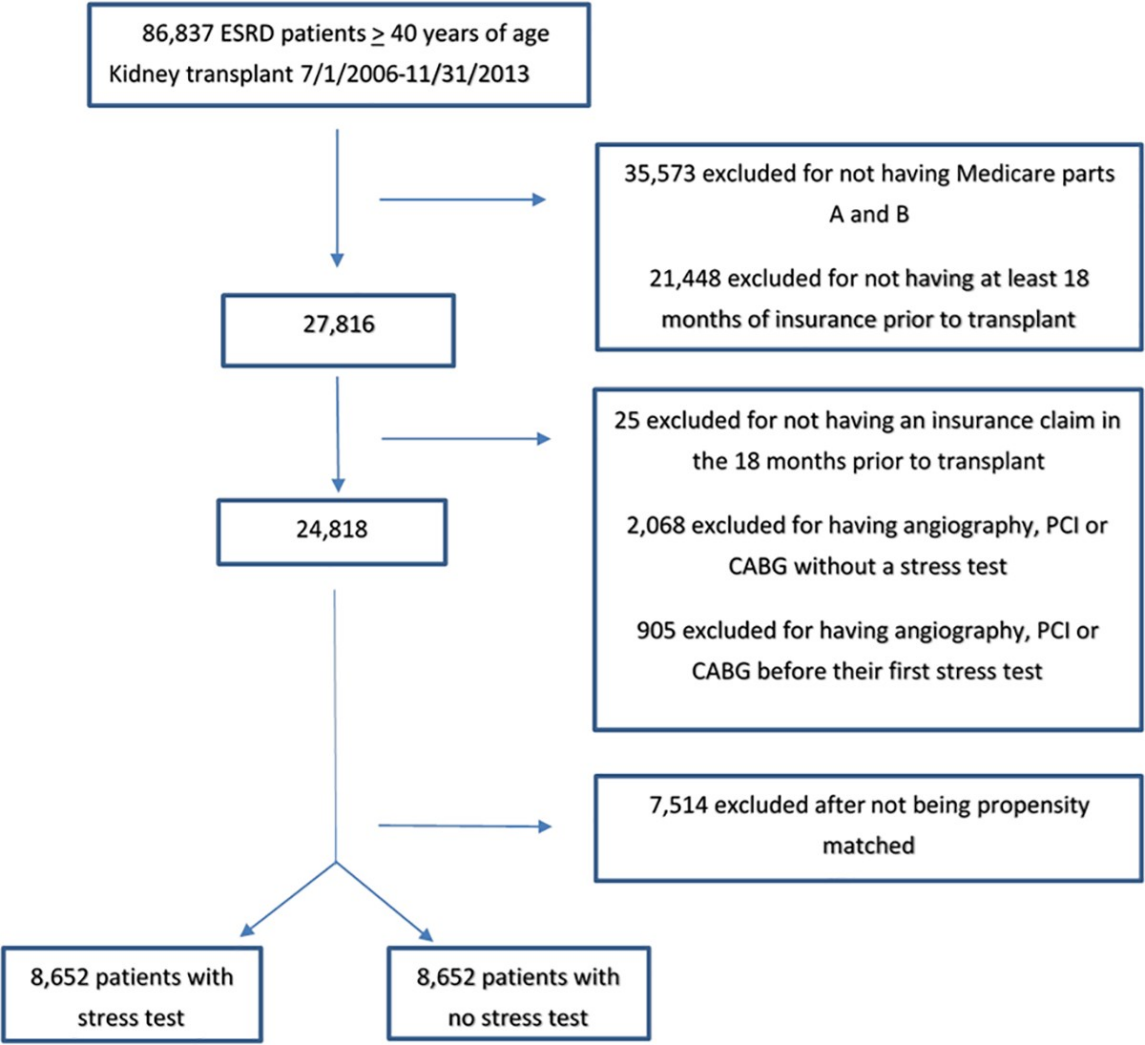
Flow diagram of study population. ESRD, end stage renal disease; PCI, percutaneous coronary intervention; CABG, coronary artery bypass graft surgery.

A total of 86,837 patients were eligible for analysis (Fig 1). 37,573 patients were excluded for having insurance other than Medicare parts A and B and 21,448 patients were excluded for not having Medicare parts A and B coverage for the entire 18 months prior to transplant. Twenty-five patients were excluded for not having any insurance claim in the 18 months prior to transplant. An additional 2,068 patients who did not undergo a stress test but who underwent angiography, PCI, or CABG in the 18 months prior to transplant were excluded, as were 905 patients who underwent angiography, PCI or CABG prior to their first stress test. After exclusions, the analytic sample included 24,818 patients of whom 14,811 (59.7%) underwent a stress test in the 18 months prior to kidney transplantation and 10,007 (39.3%) did not.

Baseline patient characteristics before matching are presented in Table 1. Patients who underwent stress testing were older (58.3. vs. 56 years, P<0.001), less often female (37.6% vs. 39%, P = 0.029), more often white (57.4% vs. 53.6%, P<0.001), and more often received their transplanted kidney from a living donor (12% vs. 9.5%, P<0.001). Patients who underwent stress testing had more risk factors for ischemic heart disease including diabetes (57.2% vs. 47.1%, P<0.001), hypertension (91.6% vs. 81.6%, P<0.001), tobacco use (19.9% vs. 16%, P<0.001) and dyslipidemia (64.5% vs. 52.2%, P<0.001). Cardiovascular disease was more common in patients who underwent stress testing including prior MI (7.2% vs. 2.5%, P<0.001), CAD (42.5% vs. 16.6%, P<0.001), chronic heart failure (24.9% vs. 15.2%, P<0.001), valvular disease (16.2% vs. 6.2%, P<0.001), arrhythmia (35.7% vs. 21.8%, P<0.001), peripheral vascular disease (25.8% vs. 16.2%, P<0.001) and cerebrovascular disease (11.2% vs. 6.6%, P<0.001).

**Table 1:** Patient characteristics before propensity matching.

	Overall (N = 24818)		No Stress Test (N = 10007)		Stress Test (N = 14811)		P value
Transplant year, No. (%)							< .001
2006	1577	(6.4%)	593	(5.9%)	984	(6.6%)	
2007	3125	(12.6%)	1200	(12.0%)	1925	(13.0%)	
2008	3183	(12.8%)	1237	(12.4%)	1946	(13.1%)	
2009	3335	(13.4%)	1294	(12.9%)	2041	(13.8%)	
2010	3346	(13.5%)	1354	(13.5%)	1992	(13.4%)	
2011	3496	(14.1%)	1454	(14.5%)	2042	(13.8%)	
2012	3391	(13.7%)	1411	(14.1%)	1980	(13.4%)	
2013	3365	(13.6%)	1464	(14.6%)	1901	(12.8%)	
Recipient Age at Transplant Time	57.34	± 9.7	56.00	± 9.7	58.25	± 9.6	< .001
Female, No. (%)	9469	(38.2%)	3900	(39.0%)	5569	(37.6%)	0.029
Race, No. (%)							< .001
Black	8929	(36.0%)	3833	(38.3%)	5096	(34.4%)	
White	13859	(55.8%)	5360	(53.6%)	8499	(57.4%)	
Other/Multi-racial	2030	(8.2%)	814	(8.1%)	1216	(8.2%)	
Height (m)	1.70	± 0.1	1.70	± 0.1	1.70	± 0.1	0.93
Weight (kg)	81.48	± 18.3	81.37	± 18.4	81.55	± 18.2	0.46
Body mass index	28.14	± 5.4	28.10	± 5.4	28.17	± 5.4	0.34
Transplant hospitalization duration in days, median (1^st^ quartile, 3^rd^ quartile)	6.0	(0.0, 522.0)	6.0	(0.0, 522.0)	6.0	(0.0, 505.0)	< .001
Donor type, No. (%)							< .001
Cadaveric/Unknown	22086	(89.0%)	9056	(90.5%)	13030	(88.0%)	
Living	2732	(11.0%)	951	(9.5%)	1781	(12.0%)	
Number of transplants performed at facility, median (1^st^ quartile, 3^rd^ quartile)	107.0	(1.0, 385.0)	100.0	(1.0, 385.0)	113.0	(1.0, 385.0)	< .001
Primary cause of end stage renal disease, No. (%)							< .001
Diabetes	8821	(35.5%)	3091	(30.9%)	5730	(38.7%)	
Hypertension	6772	(27.3%)	2893	(28.9%)	3879	(26.2%)	
Glomerulonephritis	4635	(18.7%)	2060	(20.6%)	2575	(17.4%)	
Cystic kidney/Other urologic	2053	(8.3%)	875	(8.7%)	1178	(8.0%)	
Other	2537	(10.2%)	1088	(10.9%)	1449	(9.8%)	
**Risk Factors**							
Diabetes, No. (%)	13187	(53.1%)	4709	(47.1%)	8478	(57.2%)	< .001
Hypertension, No. (%)	21634	(87.2%)	8062	(80.6%)	13572	(91.6%)	< .001
Disorders of lipid metabolism, No. (%)	14779	(59.5%)	5228	(52.2%)	9551	(64.5%)	< .001
Tobacco use, No. (%)	4552	(18.3%)	1600	(16.0%)	2952	(19.9%)	< .001
**Cardiovascular History**							
Coronary artery disease, No. (%)	7960	(32.1%)	1660	(16.6%)	6300	(42.5%)	< .001
History of myocardial infarction, No. (%)	1318	(5.3%)	253	(2.5%)	1065	(7.2%)	< .001
Congestive heart failure, No. (%)	5209	(21.0%)	1517	(15.2%)	3692	(24.9%)	< .001
Cardiomyopathy, No. (%)	1682	(6.8%)	425	(4.2%)	1257	(8.5%)	< .001
Valvular disease, No. (%)	3018	(12.2%)	621	(6.2%)	2397	(16.2%)	< .001
Arrhythmia/Conduction disease, No. (%)	7479	(30.1%)	2186	(21.8%)	5293	(35.7%)	< .001
Cardiac arrest/Ventricular fibrillation, No. (%)	243	(1.0%)	62	(0.6%)	181	(1.2%)	< .001
Pericardial disease, No. (%)	475	(1.9%)	138	(1.4%)	337	(2.3%)	< .001
Peripheral vascular disease, No. (%)	5437	(21.9%)	1618	(16.2%)	3819	(25.8%)	< .001
Cerebrovascular disease, No. (%)	2311	(9.3%)	658	(6.6%)	1653	(11.2%)	< .001
**Comorbidities**							
Chronic pulmonary disease, No. (%)	3276	(13.2%)	1119	(11.2%)	2157	(14.6%)	< .001
Liver disease, No. (%)	2012	(8.1%)	664	(6.6%)	1348	(9.1%)	< .001
Hypothyroidism, No. (%)	3496	(14.1%)	1263	(12.6%)	2233	(15.1%)	< .001

### Outcomes

Of the 14,811 patients who underwent a stress test, death within 30 days after transplant occurred in 166 (1.1%) patients who underwent a stress test and in 74 (0.7%) of 10,007 patients who did not undergo a stress test (P = 0.003). MI within 30 days occurred in 812 (5.5%) of 14,811 patients who underwent stress testing and in 355 (3.5%) of the 10,007 patients who did not undergo a stress test (P<0.001). On multivariable analysis, stress testing prior to kidney transplantation was not associated with a reduced risk of death (OR, 1.21; 95% CI: 0.90–1.62; P = 0.21), MI (OR, 1.09; 95% CI: 0.94–1.26; P = 0.25) or total MI (OR, 1.10; 95% CI: 0.61–2.00; P = 0.74).

### Propensity-matched patients

**Fig 2:**
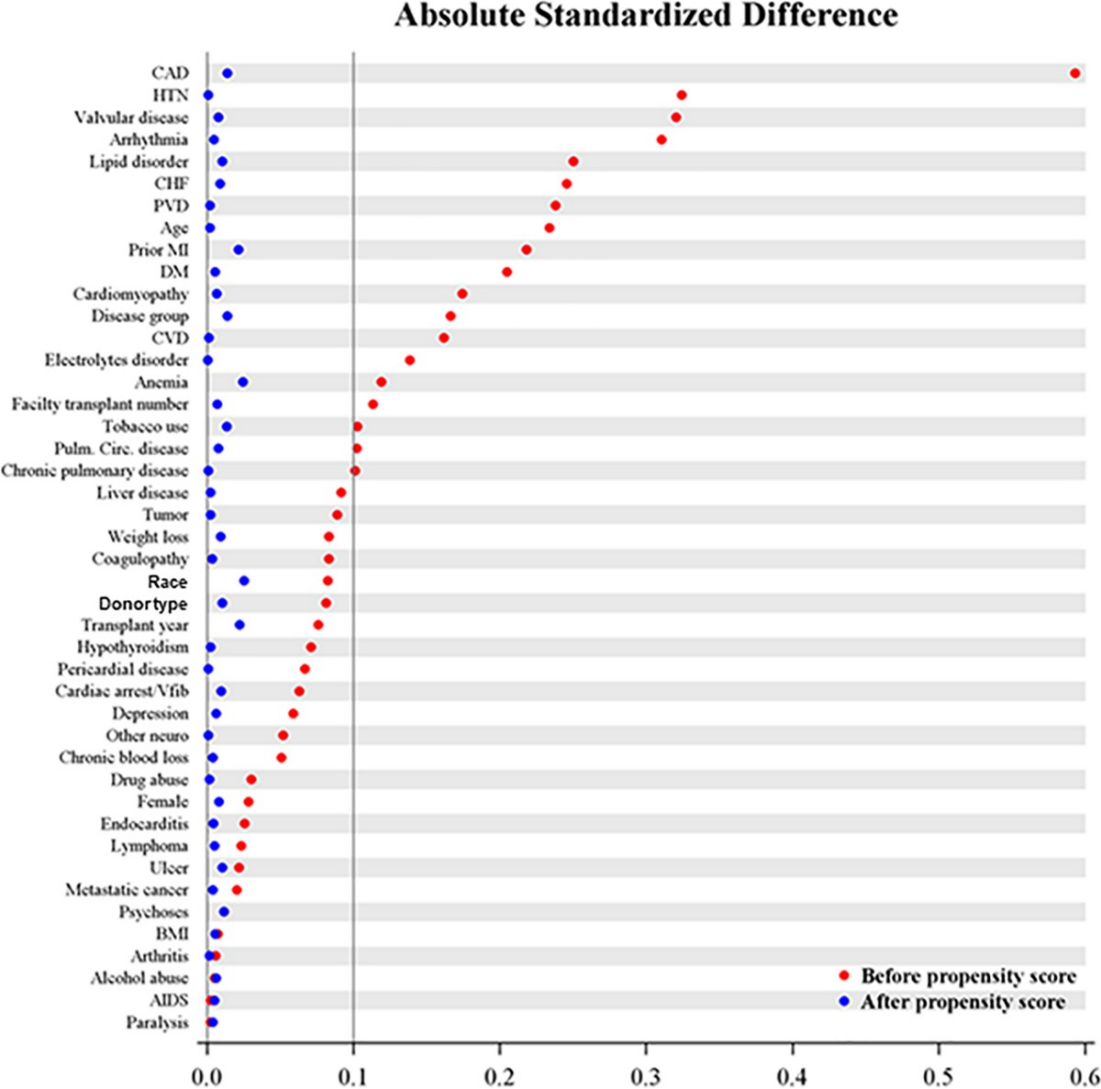
Covariate balance before and after propensity score matching. CAD, coronary artery disease; CHF, congestive heart failure; PVC, peripheral vascular disease; MI, myocardial infarction; DM, diabetes mellitus; CVD, cerebrovascular disease; Circ, circulation; VF, ventricular fibrillation; BMI, body mass index; AIDS, acquired immunodeficiency syndrome.

A total of 17,304 patients were propensity-matched in a 1:1 ratio. Baseline patient characteristics after matching are presented in Table 2. Propensity matching eliminated differences in demographics, risk factors and comorbidities between groups (Fig 2). Of propensity-matched patients who underwent stress testing, 1,149 (13.3%) subsequently underwent coronary angiography, 145 (1.7%) underwent PCI and 35 (0.4%) underwent CABG.

**Table 2:** Patient characteristics after propensity score matching.

	Overall (N = 17304)		No Stress Test (N = 8652)		Stress Test (N = 8652)		P value
Transplant year, No. (%)							0.45
2006	1045	(6.0%)	534	(6.2%)	511	(5.9%)	
2007	2085	(12.0%)	1064	(12.3%)	1021	(11.8%)	
2008	2172	(12.6%)	1092	(12.6%)	1080	(12.5%)	
2009	2232	(12.9%)	1116	(12.9%)	1116	(12.9%)	
2010	2366	(13.7%)	1173	(13.6%)	1193	(13.8%)	
2011	2502	(14.5%)	1243	(14.4%)	1259	(14.6%)	
2012	2422	(14.0%)	1198	(13.8%)	1224	(14.1%)	
2013	2480	(14.3%)	1232	(14.2%)	1248	(14.4%)	
Recipient Age at Transplant Time	56.58	± 9.5	56.59	± 9.6	56.57	± 9.5	0.89
Female, No. (%)	6846	(39.6%)	3440	(39.8%)	3406	(39.4%)	0.59
Race, No. (%)							0.16
Black	6526	(37.7%)	3221	(37.2%)	3305	(38.2%)	
White	9384	(54.2%)	4711	(54.4%)	4673	(54.0%)	
Other/Multi-racial	1394	(8.1%)	720	(8.3%)	674	(7.8%)	
Height (m)	1.70	± 0.1	1.70	± 0.1	1.70	± 0.1	0.91
Weight (kg)	81.59	± 18.4	81.53	± 18.3	81.65	± 18.6	0.65
Body mass index	28.21	± 5.4	28.19	± 5.4	28.22	± 5.4	0.73
Transplant hospitalization duration in days, median (1^st^ quartile, 3^rd^ quartile)	6.0	(0.0, 522.0)	6.0	(0.0, 522.0)	6.0	(0.0, 258.0)	0.69
Donor type, No. (%)							0.49
Cadaveric/Unknown	15595	(90.1%)	7784	(90.0%)	7811	(90.3%)	
Living	1709	(9.9%)	868	(10.0%)	841	(9.7%)	
Number of transplants performed at facility, median (1^st^ quartile, 3^rd^ quartile)	106.0	(1.0, 385.0)	103.0	(1.0, 385.0)	108.0	(1.0, 385.0)	0.22
Primary cause of end stage renal disease, No. (%)							0.86
Diabetes	5767	(33.3%)	2864	(33.1%)	2903	(33.6%)	
Hypertension	4889	(28.3%)	2440	(28.2%)	2449	(28.3%)	
Glomerulonephritis	3333	(19.3%)	1679	(19.4%)	1654	(19.1%)	
Cystic kidney/Other urologic	1503	(8.7%)	763	(8.8%)	740	(8.6%)	
Other	1812	(10.5%)	906	(10.5%)	906	(10.5%)	
**Risk Factors**							
Diabetes, No. (%)	8758	(50.6%)	4367	(50.5%)	4391	(50.8%)	0.70
Hypertension, No. (%)	15072	(87.1%)	7535	(87.1%)	7537	(87.1%)	0.95
Disorders of lipid metabolism, No. (%)	9739	(56.3%)	4847	(56.0%)	4892	(56.5%)	0.46
Tobacco use, No. (%)	3010	(17.4%)	1483	(17.1%)	1527	(17.6%)	0.37
**Cardiovascular History**							
Coronary artery disease, No. (%)	3365	(19.4%)	1659	(19.2%)	1706	(19.7%)	0.07
History of myocardial infarction, No. (%)	534	(3.1%)	251	(2.9%)	283	(3.3%)	0.13
Congestive heart failure, No. (%)	2947	(17.0%)	1459	(16.9%)	1488	(17.2%)	0.54
Cardiomyopathy, No. (%)	834	(4.8%)	411	(4.8%)	423	(4.9%)	0.67
Valvular disease, No. (%)	1253	(7.2%)	618	(7.1%)	635	(7.3%)	0.56
Arrhythmia/Conduction disease, No. (%)	4266	(24.7%)	2142	(24.8%)	2124	(24.5%)	0.72
Cardiac arrest/Ventricular fibrillation, No. (%)	127	(0.7%)	60	(0.7%)	67	(0.8%)	0.53
Pericardial disease, No. (%)	271	(1.6%)	136	(1.6%)	135	(1.6%)	0.95
Peripheral vascular disease, No. (%)	3117	(18.0%)	1562	(18.1%)	1555	(18.0%)	0.88
Cerebrovascular disease, No. (%)	1265	(7.3%)	634	(7.3%)	631	(7.3%)	0.93
**Comorbidities**							
Chronic pulmonary disease, No. (%)	2078	(12.0%)	1038	(12.0%)	1040	(12.0%)	0.96
Liver disease, No. (%)	1259	(7.3%)	627	(7.2%)	632	(7.3%)	0.88
Hypothyroidism, No. (%)	2325	(13.4%)	1166	(13.5%)	1159	(13.4%)	0.88

Outcomes for the propensity-matched cohort are presented in Table 3. Death within 30 days occurred in 72 (0.83%) patients who had a stress test and in 65 (0.75%) patients who did not have a stress test (OR 1.07; 95% CI: 0.79–1.45; P = 0.66). MI within 30 days occurred in 339 (3.9%) patients who had a stress test and in 333 (3.8%) patients who did not (OR 1.03; 95% CI: 0.89–1.21; P = 0.68). Fatal MI occurred in 17 patients who underwent a stress test (0.20%) and in 15 patients who did not (0.17%) (OR 0.968; 95% CI: 0.71–1.32; P = 0.84). In total, of the 672 patients who developed an MI, 32 (4.8%) subsequently died within 30 days. Of the 137 patients who died within 30 days of their transplant, only 32 (23%) had suffered an MI.

**Table 3:** Outcomes in propensity-matched cohort with and without stress testing.

Outcome	Stress Test (N = 8652)	No Stress Test (N = 8652)	Odds Ratio (stress vs. no stress)	95% Confidence Interval	P value
Death within 30 days after transplant	72 (0.83%)	65 (0.75%)	1.07	(0.79, 1.45)	0.66
Acute myocardial infarction within 30 days after transplant	339 (3.9%)	333 (3.8%)	1.03	(0.89, 1.21)	0.68
Fatal myocardial infarction	17 (0.20%)	15 (0.17%)	0.97	(0.71, 1.32)	0.84

## Discussion

The significant findings of this observational cohort study of cardiac stress testing in ESRD patients after placement on the waiting list but prior to kidney transplantation are two-fold. First, patients who are placed on the waiting list for kidney transplantation are at relatively low risk of perioperative death, MI or fatal MI. Second, and most importantly, cardiac stress testing in the 18 months prior to renal transplantation is not independently associated with a reduction in 30-day death, MI or fatal MI after adjustment for differences in demographics and comorbidities by logistic regression and propensity matching techniques.

The role of preoperative cardiovascular risk assessment is to assist the patient and health care providers in weighing the benefits and risks of surgery. Given the unpredictability of donor availability and the timing of transplant surgery, many patients on the kidney transplant waiting list undergo a version of preoperative risk assessment whereby they undergo periodic stress tests to monitor them for the development of ischemia. That the stress tests were intended for screening as opposed to investigation of ischemic symptoms is supported by the low rate of coronary angiography (13.3%) and revascularization (2.1%) in the propensity matched cohort. Screening stress tests are not recommended in other preoperative patient populations. The 2014 American College of Cardiology/American Heart Association (ACC/AHA) guideline on perioperative cardiovascular evaluation and management of patients undergoing noncardiac surgery recommends pre-operative cardiac evaluation, including stress testing, only if it would be indicated in the absence of the upcoming surgery [10]. Thus, in general, cardiac stress testing in anticipation of surgery should not be performed. However, ESRD is considered a significant risk factor for CAD and such patients undergoing kidney transplantation are considered at increased risk for perioperative adverse cardiac events. A 2016 study of United States hospital admissions from 2004 to 2013 found a 3% incidence of major adverse cardiovascular and cerebrovascular events (in-hospital, all-cause death, acute MI, or acute ischemic stroke) in the postoperative period [11]. These events were most common after vascular (7.7%), thoracic (6.5%), and transplant surgery (6.3%). In recognition of the increased risk of patients undergoing kidney transplant surgery, a 2012 AHA Consensus Statement, in the absence of any high-quality data, indicated that stress testing could be considered in kidney transplantation candidates with no active cardiac conditions based on the presence of multiple CAD risk factors regardless of functional status (Class IIb, Level of Evidence C) [2]. However, the current results suggest that patients with ESRD who have been screened for ischemia prior to placement on the waiting list and then undergo kidney transplantation are not at particularly high risk, perhaps because the highest risk patients are removed from consideration of transplant during the initial screening process. In the Coronary Artery Revascularization Prophylaxis (CARP) trial of patients who underwent surgery for peripheral arterial or aortic disease, 30-day mortality was 3.2% [12] as opposed to 0.8% in the propensity-matched cohort and 1.1% in the stress testing arm of the overall cohort in the current study. Furthermore, the fatal MI rate of 0.2% makes it highly unlikely that any screening strategy could be shown to reduce that rate further.

Many studies have demonstrated that stress testing can further stratify the risk of adverse perioperative events [10–21]. Although there is a clear relationship between the degree of myocardial ischemia detected and prognosis, there is no evidence that prophylactic revascularization prior to surgery improves outcomes [1, 22–30]. However, it is recommended that ESRD patients with ischemia on stress testing be referred for coronary angiography and revascularization, if technically feasible, to maintain their candidacy for transplant [31]. Data from CARP indicates that prophylactic revascularization does not improve outcomes in a vascular surgery population at 4-fold greater risk of death than ESRD patients undergoing kidney transplantation. In that randomized, controlled trial, PCI was performed in 59% and CABG was performed in 41% of patients assigned to prophylactic revascularization. At 2.7 years of follow-up, mortality in the revascularization group was 22% versus 23% in the no-revascularization group (relative risk, 0.98; 95% confidence interval, 0.70 to 1.37; P = 0.92). Within 30 days of the vascular operation, 3.1% of patients assigned to revascularization and 3.4% of patients not assigned to revascularization died (P = 0.87), whereas MI occurred in 12% of the revascularization group and 14% of the non-revascularization group (P = 0.37) [12].

The only prospective, randomized data on pre-operative revascularization in ESRD patients awaiting transplant comes from the 1992 study by Manske et al. who randomly assigned 31 transplantation candidates with insulin-dependent diabetes mellitus and CAD (>75% stenosis) to revascularization or medical therapy with a calcium channel blocker and aspirin [32]. Ultimately, 10 of 13 medically managed and 2 of 13 revascularized patients experienced the composite endpoint consisting of unstable angina, MI or cardiac death. Given the small sample size of exclusively diabetic patients, the high event rate among the medically managed group and advances that have occurred in the medical management of diabetes and ischemic heart disease, including the use of angiotensin-converting enzyme inhibitors and statins, the findings of this study are no longer applicable to contemporary practice.

There are several limitations of this study. First, although our list of confounders was extensive, propensity analyses cannot account for selection bias related to unmeasured characteristics. It should be noted however that a randomized clinical trial is unlikely to be performed to address the value of preoperative stress testing because the low event rates would require sample sizes too large to be feasible. Second, this study is based on administrative data including ICD codes that were designed for reimbursement, not clinical phenotyping. Third, administrative coding for MI does not distinguish between type I (plaque rupture) and type II (supply-demand mismatch) MI. These different types of MI are approached with different diagnostic and therapeutic strategies and have different prognostic implications that may influence the results of this study. In addition, there was no uniform practice to screen patients for the development of postoperative MI. Fourth, we cannot determine how many patients underwent stress testing during screening for placement on the waiting list and were never listed for transplant due to detection of extensive CAD. However, our intention was specifically to evaluate the role of stress testing after listing and not during the evaluation process for placement on the waiting list. In addition, since this data set does not provide specific reasons for removal from the waiting list, we cannot ascertain if the results of surveillance stress testing led to the removal of any patients from the waiting list. Finally, since follow-up was limited to 30 days, the conventionally defined perioperative period, the possibility of late benefit related to stress testing cannot be excluded.

In conclusion, the routine performance of cardiac stress tests on ESRD patients in the 18 months prior to kidney transplantation is not associated with a reduction in postoperative death, MI or fatal MI. The use of routine cardiac stress tests in this population should be reconsidered until a clinical benefit is demonstrated in randomized, controlled trials.
